# Supplementary material for: Chromothripsis during telomere crisis is independent of NHEJ, and consistent with a replicative origin
Source: Genome Res. 2019 May;29(5):737–49. doi: 10.1101/gr.240705.118 (PMC6499312; doi:10.1101/gr.240705.118)
Supplement: Supplemental Material [file supp_gr.240705.118_Supplemental_file_1.zip › contigs/annotated_contigs/DB79/contig.2.DB79_length_533_mean_cov_7.45590994371.docx]

**DB79_length_533_mean_cov_7.45590994371**

```
TTTTTTAGCTTCAAGGAGACTGCCCAGAAGACTGCCAACAATTCTTTCCCTGCCTTGGCCTACAACTACATGCATTCCATTCGGAAACT
 >chr22:18209228-18209394 + E=3e-67 p=0e+00
ATGTAGGTTTAAACAGTGTTTCATTCCTAGAACTTCAGTTTAGGAAAGAATTTGCTTTCCAGATTGATGTTTATGTT|CGAGA|CCAGA
                                                                                   >chr
CTGGTCTCGAACTCCTGACCTCGTGATCCGCCTGCCTTGGCCTCCCAAAGTGCTTCTACAAAAGCTTTAGGTGATGGTCCCGCGTCATA
22:18130082-18130444 + E=2e-206
ATTACCTTTCATCCAATCTAGCTTTTTTTTCCCCCCGTCCATTCTCAAAACTGACAAATTTGCTTCTTTCAGTACTCTGGATCTGTGAT

ACTACAAAAACCACCTTGTTTTGCTACCGTATCCTTCCGTATTACTAGTGTTAGTGTTTTCACCTCTGCATTCCTCTCACCTAGTACAT

TACCTGGCATATAGTAAATATGTGTTGAATAAATACAGTTAAGGTCTAAGAGGAAGTGAAGACGGTGTACCCAATTAAGTGGTTGTGCT

G
```
